# Healthcare access as a right, not a privilege: a construct of Western thought

**DOI:** 10.1186/1747-5341-2-2

**Published:** 2007-03-28

**Authors:** Thomas J Papadimos

**Affiliations:** 1 Departments of Anesthesiology, Medicine, and Medical Microbiology and Immunology University of Toledo College of Medicine 3000 Arlington Avenue Toledo, Ohio 43614, USA

## Abstract

Over 45 million Americans are uninsured or underinsured. Those living in poverty exhibit the worst health status. Employment, education, income, and race are important factors in a person's ability to acquire healthcare access. Having established that there are people lacking healthcare access due to multi-factorial etiologies, the question arises as to whether the intervention necessary to assist them in obtaining such access should be considered a privilege, or a right. The right to healthcare access is examined from the perspective of Western thought. Specifically through the works of Aristotle, Immanuel Kant, Thomas Hobbes, Thomas Paine, Hannah Arendt, James Rawls, and Norman Daniels, which are accompanied by a contemporary example of intervention on behalf of the medically needy by the The Johns Hopkins Urban Health Institute.

As human beings we are all valuable social entities whereby, through the force of morality, through implicitly forged covenants among us as individuals and between us and our governments, and through the natural rights we maintain as individuals and those we collectively surrender to the common good, it has been determined by nature, natural laws, and natural rights that human beings have the right, not the privilege, to healthcare access.

## Background

The health of people in different socioeconomic strata is disparate whether measured by education, type of employment, or income level [1], but those living in poverty exhibit the worst health status [2,3]. In the United States there are over 45 million uninsured or underinsured people who by definition have restricted access to healthcare [4]. Although not all of them are unemployed, or underemployed, there is no doubt that occupational status affects insurance status, which affects health status, and there is convincing evidence that low socioeconomic status causes poor health [5], as does race [6].

Having established that there are people who have a lack of healthcare access due to multi-factorial etiologies the question arises as to whether the intervention necessary to assist them is something that should be considered as a privilege, or a right. I will posit in this dialectic that access to healthcare is a right, not a privilege.

## Discussion

### Aristotle and the soul

Many have argued on behalf of the existence of "the soul", a concept perpetuated in many religions and philosophies. Aristotle (384–322 B.C.E) hypothesized the existence, content, and necessary conditions of the soul [7]. His arguments as to the existence of a soul, and what it is, will serve as the initial kernel in the examination of access to healthcare as a right.

Aristotle argues that there are natural bodies, some are living and others are not. In his argument Aristotle calls attention to the fact that whatever has a soul, in the human sense, displays life. The soul is the first entelechy (the real existence of a thing; not merely its theoretical existence; in some philosophies it is a life-giving force believed to be responsible for the development of all living things). Aristotle believed that a besouled natural body could grow, decay and at the same time provide itself with nutrition; the soul is further characterized by the powers of sensation (that allow pain and pleasure, which lead to desire), thinking (besouled beings have the power of thought, calculation, and imagination), and motivity [7]. The soul is a substance that is the definitive essence of a thing. Aristotle claimed soul and body were not the same, "the body cannot be soul; the body is subject or matter, not what is attributed to it. Hence there must be a substance in the sense of the form of a natural body having life potentially within it" [7]. Aristotle illustrates the relationship of the body to the soul by remarking that:

"If the whole body was one vast eye, sight would be it soul. As the eye is a tool for seeing with, but a living tool which is part of ourselves, so the body is like a tool or instrument for living with. Hence we may say of the soul that it is the 'end' of the body, the activity to which the body is instrumental, as seeing the 'end' to which the eye is instrumental" [8].

Putting aside the concepts of good and evil, and having determined that souls need nourishment, have sensation (pain, pleasure, desire) and that they can think, calculate, and imagine, would it not be right and prudent to assist this entity called "soul" (that moves through a society by using a body which needs nutrition/nurture to slow or prevent its decay) to endure and persist as long as possible in the midst of others like itself? To press the argument further, would humankind not be better off if the vehicle of social interaction (body with soul) was cared for and nourished, not only by itself, but assisted and nurtured by other such vehicles in a society? Would there not be an alteration in its growth, perception, calculation and imagination that resulted in a higher probability of positive (good) consequences or actions?

For the soul to be fulfilled it must be nourished and grow. Therefore, healthcare access is necessary for the soul to attain its fullest growth, to nurture its intellect as a thinker and a citizen. While the soul is the first entelechy, good health is needed to reach the final entelechy, "a life of intelligence and character actively functioning" [8].

Theology, politics and economics aside, respecting, nurturing, and caring for human beings and their "essence", whatever that may be, can only help a society grow and prosper. To lead an "intelligent life" and to be an "actively functioning" member of society a body, whether one believes Aristotle's arguments about souls pertain to it or not, needs access to healthcare.

### Kant and the force of morality

Aristotle argues that for a soul to exist and to grow it needs nourishment to allow it to think (imagine), sense (pain and pleasure), and desire (thus we have a human being). Everyone on earth, according to Immanuel Kant (1724–1804), "exists as an end in himself, not merely as a means for arbitrary use by this or that will: he must in all his actions, whether they are directed to himself or to other rational beings, always be viewed at the same time as an end" [9].

The existence of humans is not for their use only as a means to accomplish a task. We are not, "a means to someone else's pleasure or well-being and our personhood consists in our status as a rational agent of worth" [9]. If people should not be treated as things, then it must be recognized that all people have an absolute worth simply because they exist, therefore you must "act in such a way that you always treat humanity whether in your own person or in the person of any other, never simply as a means, but at the same time as an end" [9]. This statement has been called the Formula of Humanity [9]. Kant explains that society depends upon interactions between people, we all serve each other's interests at one time or another, but we are never to be treated only as a means. Everyone has worth.

If every human needs nourishment, thinks, senses, and has an absolute value (worth), and if Kant's categorical imperative addressing people as an end-in-themselves is accepted (the Formula of Humanity), then we have established a force of morality in that: (1) all living natural bodies having needs, sensations/desires, and intelligence are thus human, and (2) as natural bodies they (humans) have an absolute worth that makes it necessary that all humans treat each other as one wishes to be treated. This moral force is irresistible.

In regard to this force of morality (as it concerns people as ends-in-themselves) Kant claims:

"The motive of morality is quite different from that of interest or desire. It rules us absolutely and necessarily; we feel its power even when we are most defying it. It is not one consideration to be balanced against others, but rather a compelling dictate that can be ignored, but never refuted" [10].

We can politically ignore the force of morality, but it will always be there. The question of healthcare access for all people will be a persistent moral and politico-economic interrogative.

### Hobbes and covenants

We have established that natural living bodies (humans) should be treated as ends-in-themselves (all persons have absolute value, or worth). Humans have worth that must be preserved. This preservation of life can be argued to be sacred, or important, whether it is viewed religiously, socially, economically, or militarily. Religions universally value the preservation of life and the soul from the perspective of immortality. Socially, we need preservation of life so that we have like beings with which to interact. Economically, we need taxpayers and workers. Even the military places an extremely high value on its personnel because of the costs of training and the necessary numbers needed for its interventions. Thomas Hobbes (1588–1679) felt that reason would allow man to figure out what must be done to preserve life. To this end, in his epic work, Leviathan, he presents his rules, or Laws of Nature, and explains, "A law of nature (lex naturalis) is a precept or general rule, found out by reason, by which a man is forbidden to do that which is destructive of his life or taketh away the means of preserving the same, and to omit that by which he thinketh it may be best preserved" [11].

Hobbes reasoned that every person has a "Right of Nature" in that, "every man has a right to everything, even to one another's body" [11]. In other words, anybody can do anything. However, as soon as we come to our senses we realize there are laws of nature that rein in this liberty. These laws that are put into place give us the concept of "obligation".

Hobbes presents three important laws of nature that cause us obligation in regard to our actions. Hobbes' laws involve (1) seeking peace, (2) laying down the right of nature and making covenants, and (3) performance of covenants. In following Hobbes' laws to their conclusion an argument can be made that a covenant exists between members of a society, though unwritten, to provide security or well being for one another. Furthermore, since "we the people" are the government, such a covenant may actually exist between the members of a society and the government as to the provision of access to healthcare.

Hobbes' first law is to seek peace, "that every man, ought to endeavor peace, as farre as he has hope of obtaining it; and when he cannot obtain it, that he may seek, and use, all helps, and the advantages of warre" [11]. He follows this statement with the advisory that society should "seek peace, and follow it", and if a society cannot get peace, they must defend themselves.

In regard to healthcare, seeking peace and defending one's self can be manifested in the form of negotiations and the use of the electoral process to gain a favorable hearing on the argument that healthcare is a right to be shared by or available to all. However, this becomes difficult for the uninsured or the underinsured to pursue because they are without economic strength, many times they are racial minorities and can be marginalized by the political process.

Hobbes' second law, "lay down the right of nature", also applies and is extremely important. Here Hobbes indicates that we should lay down our right "to do anything" if others are willing to do the same. The rule "requires each of us to be satisfied with as much liberty toward other human beings as we are willing to allow them with respect to ourselves" [12]. When we give up liberty (or portions there of) we can:

"Renounce this right or transfer it to someone else. In engaging in either of these acts, we are removing an impediment to someone else's use of those same things or someone else's exercise of their right to them. In this way we are bestowing benefits on these other people. If we care not who receives the benefit, we are said to renounce our right; if we wish the benefit to accrue to some one or more particular persons, we are to speak of transferring our right to them, which amounts to a gift. We may, then, either abandon our right or give it away to other people" [12].

Whether a right is renounced or transferred a promise is made (commitment) and thus an obligation is incurred (by us). Hobbes considers the obligation created by "laying down the right of nature" as the origin of morality [12]. Nature does not require commitment, i.e., one can do whatever he or she pleases (there is no morality in nature according to Hobbes). However any commitment to an ideal, or situation, incurs an obligation on my part. Thus there is justice in my promise of commitment and injustice is my breaching of a promise of commitment [12]. The "implication" here is that in "laying down the right of nature" all members of a society obligate themselves to one another, to live in peace among one another, and thus to provide mutual security for one another.

This second law leads to the concept of contracts, or covenants. When people renounce or transfer the "right of nature" a contract, or covenant, is made. Covenants may "not be explicitly conveyed and acknowledged by words. The sign or indication of a contract may be by inference, and be implicit, or covert" [12]. Hobbes claims that, "generally a signe by inference of any contract is whatsoever sufficiently argues the will of the contractor" [11]. The "right" at stake here is the right of the medically needy to "transfer" their need (per Hobbes' theory) for healthcare to a society in which all of its members have pledged mutual cooperation by agreeing to Hobbes' second law. This acquiescence to Hobbes' second law has in fact occurred in our society although it seems not to have been acknowledged. Applying Kant's thoughts to this condition, this implicit covenant may be ignored politically, but it has a force of morality that cannot be refuted. Even though Hobbes thinks that the moral life is not constitutive of human nature [12], he would probably agree with Kant's thoughts on the basis of social, political, and economic reasoning.

Hobbes' third law regards the performance of covenants. When a covenant is made it is important that "men performe their covenants made: without which, covenants are in vain, and but empty words, and the right of all men to all things remaining, wee are still in the condition of warre" [13]. If we have agreed to live in mutual security then we must make good on our promises, implied or otherwise.

The concept of "inference" or "implicitness", regarding Hobbes' second law, and the obligation of performance of covenants regarding the third law are very important. The citizens of the United States have come to hope and expect availability of healthcare access. The growing number of persons that are uninsured, or underinsured, have a limited access to prompt and adequate healthcare. The provision of healthcare is an implied covenant that the medically needy have hoped and expected society to embrace.

### Paine and the rights of man

Hobbes spoke of natural laws. Thomas Paine (1737–1809) spoke of natural rights [14]. Such rights are those that pertain to a person in relation to his or her right to exist. These rights are of two kinds: the first kind regards intellectual rights (rights of the mind, including religion), and the second involves a person's right to pursue comfort and happiness as long as the rights of others are not injured.

These natural rights act as a foundation for civil rights. A member of society does not have enough individual power to pursue these rights (civil), which are all those that relate to security and protection. Therefore, natural rights are:

"Those in which power to execute is perfect in the individual as the right itself... The natural rights that are not retained, are all those in which, though the right is perfect in the individual, the power to execute them is defective. They answer not his purpose. A man, by natural right, has a right to judge his own cause; and so far as the right of the mind is concerned, he never surrenders it: but what availeth it him to judge, if he has not power to redress? He therefore deposits this right in the common stock of society, and takes the arm of society, of which he is a part, in preference and in addition to his own" [15].

From this line of reasoning Paine concludes that all civil rights grew out of natural rights; that civil power is an aggregation of people's natural rights and this is so because the individual is ineffective to pursue security and protection alone, but together the will of the many is "competent to the purpose of every one" [15]; and that this imperfect individual ability to secure collective security, when given to a central power (government), cannot be used to disarm a person of their natural rights. Paine concludes that, "Every man is a proprietor in society, and draws on the capital as a matter of right (authors' emphasis)" [15].

In view of the aging baby boomer generation, shrinking urban tax bases, and projected shortages in skilled labor there is some reason to think that Paine would consider healthcare access in today's society a matter of security, personal and national. All people in a society today surrender their natural rights to a central power. Every person expects security and benefit from the common good, thus an argument can be made that individuals may draw on the capital of collective security in regard to healthcare access, "as a matter of right" [15].

### Arendt and the human condition: speech, action, power and the space of appearance

The Human Condition (1958) by Hannah Arendt (1906–1975) is as relevant now as it was at the time of its publication [16]. Her thoughts and process of reasoning are insightfully cutting and are applicable to the topic of the process of acquiring healthcare access.

Humans, having absolute worth, having made an implicit covenant with one another for their mutual good, and having surrendered their natural rights to a central power for their collective security, must by definition be equals, and in addition to being equals, they are distinct:

"Human plurality, the basic condition of both action and speech, has the twofold character of equality and distinction. If men were not equal, they could neither understand each other and those who came before them nor plan for the future and foresee the needs of those who will come after them. If men were not distinct, each human being distinguished from any other who is, was, or will ever be, they would need neither speech nor action to make themselves understood. Signs and sounds to communicate immediate, identical needs and wants would be enough" [17].

Humans' speech and action make them distinct, "Through them, men distinguish themselves instead of being merely distinct; they are modes in which human beings appear to each other, not indeed as physical objects, but qua men" [17]. This distinctness rests on what people say (speech) and what initiative they exhibit (action). Some forms of speech make people better known and some forms of action make some people rich and others poor, disabled, or even dead. Such variety among humans does cause "distinctness", but it does not change equality.

All humans are equals, all humans are different (distinctive), and all humans are heroes. However, heroes have no distinctive qualities; "the word 'hero' originally, that is, in Homer, was no more than a name given each free man who participated in the Trojan enterprise and about whom a story could be told" [17]. Courage, something we identify with a hero, according to Arendt, is nothing more than being willing to act, speak, and insert one's self into society and to begin one's own "story". Arendt's concept of courage has nothing to do with suffering; it has everything to do with:

"Leaving one's private hiding place and showing who one is, in disclosing and exposing one's self. The extent of this original courage, without which action and speech and therefore, according to the Greeks, freedom, would not be possible at all, is not less great and may even be greater if the 'hero' happens to be a coward" [17].

In other words, a hero is a human being who awakens each morning and inserts himself, or herself, into society. All men and women are therefore heroes; all are equal, yet distinct. They may vary in height, weight, race, wealth, sex, age, or religion, etc., but they all are heroes who insert themselves into the world and begin their story.

The space of appearance is "the space where I appear to others as others appear to me, where men exist not merely like other living or inanimate things but make their appearance explicitly" [17]. The space of appearance is not like a room or a building, it comes into being when people interact in speech and action and it disappears with the dispersal of people, such as with wars and natural disasters [17]. It is a public space that endures through the rule of law and people sharing their lives together. It is the place where speech and action come to bear upon society. It can also be called the public realm.

Power is an actuality that occurs between people when they speak and act together, "Power is what keeps the public realm, the potential space of appearance between acting and speaking men, in existence" [17]. It too disappears when people disperse. Power cannot be held in reserve like tanks and weapons of mass destruction, it exists only when actualized by men and women. Power is a potential and is actualized where words and actions, "have not parted company... where words are not empty and deeds not brutal, where words are not used to veil intentions but to disclose realities, and deeds are not used to violate and destroy but to establish relations and create new realities" [17].

The medically needy are usually those of a disadvantaged socioeconomic status [2,3,5,6]. To actualize the power referred to above the medically needy would have to interact in the space of appearance. The space of appearance cannot be occupied by all of the people all of the time, but some people, such as the medically needy, may never enter it:

"This space does not always exist, and although all men are capable of deed and word, most of them–like the slave, the foreigner, and the barbarian in antiquity, like the laborer or the craftsman prior to the modern age, the jobholder or businessman in our world–do not live in it. No man, moreover, can live in it all the time. To be deprived of it means to be deprived of reality, which, humanly and politically speaking is the same as appearance" [17].

There are times when others may need to come forward and speak for those who have no power and cannot enter the space of appearance; even better, they should secure the entrance of the powerless so that they may be heard, "To men the reality of the world is guaranteed by the presence of others..." [17]. Every human must be able to partake of the space of appearance at some time (and thus the sharing of power), even though he or she cannot partake of it at all times. To deny this is to deny the soul of its nutrition, morality of its force, and the existence of any covenant of, or right to, mutual security.

### The presence of others: Rawls and Daniels and the application of justice to healthcare

While navigating the pathway of healthcare access as a right emphasis has been placed upon Aristotle's view of the soul, Kant's regard for the absolute worth of humans, Hobbes' position on covenants, Paine's support of rights (both intellectual and in the pursuit of happiness), and Arendt's eloquence on the human condition. Now a juncture in the argument is reached where Arendt's desire for others to come forward and speak for those who have no power is addressed. John Rawls (1921–2002) and Norman Daniels have championed justice and its application to healthcare access.

John Rawls' work on "Justice as Fairness" [18] and Norman Daniels' framing of the argument for universal healthcare by using Rawls' principles to demonstrate that there are social determinants of health affecting the health achieved by societies [19,20], complete a Kantian thread that runs throughout this dialectic.

John Rawls believed that,

"Each person possesses an inviolability founded on justice that even the welfare of a society as a whole cannot override. Therefore in a just society the rights secured by justice are not subject to political bargaining or the calculus of social interests" [18].

While Rawls' work was not directly related to healthcare access it provided principles that Daniels uses to illuminate the "just distribution of the social determinants of health" [20]. Rawls argued two principles. The first principle addressed an equal claim by all citizens to equal rights and freedoms. The second principle was in two parts. The first part of the second principle states that there must be equality of opportunity provided by social structures so all citizens may have the same chance of gaining income, wealth, position, and social advantages. The second part of the second principle states that inequalities will be tolerated only when such inequalities work to the advantage of society's most disadvantaged [18].

Daniels emphasizes that people in wealthy countries live longer and that within a particular country's social structure, government policies and national/regional cultures contribute to health variation within each country. He further argues that a country's health depends not only on its economy, but how the wealth is distributed. Wealthy countries may demonstrate better health than poorer countries, but wealthy nations also demonstrate variability in health along lines of income distribution. This variability within a country, or socioeconomic gradient, can be identified throughout all ranges of income distribution. Additionally, Daniels explains that what is invested by a country/government in its society, education for example, benefits the health of a nation. This not only varies from country to country, but in the United States it varies from state to state. Thus politics and public policy begin to play large roles. Investments in education, the work place, health and immunizations, and nutrition, can provide a major advantage to those in need. In other words, health inequities need to be addressed through the correction of economic inequities. So while the United States engages in a debate on healthcare reform, interested parties need to understand that while there may be a "focus on healthcare at the point of delivery", the battle for healthcare access may also need to be addressed along the lines of the social determinants of health.

### Academic Medical Centers (AMCs): a space of appearance for power and action

The reality of this world, indeed, is guaranteed by the presence of others. While "others" have provided the theoretical basis for healthcare as a right, and "others" have been political proponents of such a right, what vehicle can be the conduit for championing such a right? The natural conduit for providing access to healthcare is the AMC; the necessary space of appearance can be provided by AMCs. Why should AMCs champion healthcare access, and what role should they play?

Historically, AMCs have been at the epicenter of providing healthcare for the disadvantaged. Eli Ginzberg's excellent treatise, "Teaching Hospitals and the Urban Poor", provides an enlightening historical review [21]. Many well-known AMCs established themselves in the 19^th ^century. These centers were a union of teaching hospitals with medical schools established through philanthropy. They provided care to those in the neighborhood that were ill, specifically to those urban residents affected by intermittent epidemics that plagued population centers. The vast majority of these patients were poor. The acute care hospital was not used by the middle and upper classes until the very late 19^th ^and 20^th ^centuries.

AMCs have treated a disproportionate number of the uninsured for years. There is a Kantian force of morality regarding the historical mission of AMCs. This backdrop of the health plight (urban and rural) does not fade easily; it comes to the foreground. It is an irresistible moral force. This force of morality, as mentioned previously, can be ignored, but never refuted [10]. AMCs are further championed in this dialectic because of their (1) locations (usually urban), (2) expertise (subspecialty, research, and otherwise), and (3) educational mission (training of physicians, nurses, researchers, and allied health providers) [21]. Therefore, from a historical, geographic, expert, educational, and moral perspective AMCs are naturally selected conduits to argue for a right to access healthcare.

However, AMCs cannot be expected to shoulder the brunt of health needs of urban and rural America without financial assistance. Such help must come from local, state, and federal governments. Unfortunately, such an outlay of resources needs more than a moral will, it needs a political will that is increasingly difficult to muster in the face of the extended conflicts in Iraq and Afghanistan and the continued trade deficit and national debt of the United States. With a limited source of funding, what should be provided and how?

AMCs treat many uninsured patients in their emergency departments. The emergency department for the uninsured and underinsured is not an ideal field in which to address the order of battle when combating the problem of healthcare access. Intervening early in the course of an illness is better, but ideally, disease prevention through the modification of social determinants that may be initiators of disease, including early access to healthcare, is a more worthy course of action for AMCs.

A noteworthy example of an AMC's attempt to address the need for healthcare among the socially disadvantage was the establishment of The Johns Hopkins Urban Health Institute. It "was established with the mission to marshal the resources of the university and external groups to improve the health and well being of the residents of East Baltimore" [3]. While the United States has been a country of medical breakthroughs and advancements the vulnerable segments of its society have experienced inadequate delivery of healthcare [3,22].

Urban health in East Baltimore had been extremely poor in the 1990's. Besides poverty and violence (near the top in the USA) this part of the city had 12% of its first graders with asthma, 18% of all its births had complications secondary to low birth weight, sexually transmitted disease rates that were the highest in the country, and a rate of syphilis that was the highest of any city in the developed world [3]. There were also very high rates of diabetes, human immunodeficiency virus, substance abuse, pulmonary, cardiovascular, and cerebrovascular disease [3].

In 1999 several working groups determined there were 13 obstacles to improving the health of East Baltimore: poverty; a drug-based economy; abandonment of the economically mobile to the suburbs; lack of education; lack of economic and political power; economic and environmental pressures that divert people from health lifestyles; issues of race and discrimination; lack of continuity of care; lack of community health workers; lack of health insurance; mistrust of the AMC, government and the local community; a lack of coordination between research projects and clinical care; and concerns about privacy of health information [3]. The institute was established soon after these obstacles were identified (2000). Its three strategic goals were, (1) strengthening and enhancing urban health research and learning both locally and nationally, (2) reducing disparities in health and healthcare for East Baltimore residents, and (3) promoting economic growth in the area. Goal 1 was addressed through university support for community-based research. Funding of projects such as "The Amazing Grandmothers", support services to grandmothers who cared for children whose parents were no longer caring for them because of drug abuse; and "Spirituality, Substance Abuse, and Mental illness", a service improvement project for those with co-existing substance abuse disorders and mental illness. Goal 2 was an active practice of medical intervention against HIV/AIDS through testing, counseling, and placement in appropriate care; and the development of a local, university supported primary care system aimed at: (a) impacting East Baltimore health, (b) encouraging community involvement, (c) educating university medical staff in urban health, (d) decreasing emergency department visits and the need for tertiary care, and (e) providing evidence-based proof that primary care can improve the health of urban citizens. Goal 3 was promoted by providing East Baltimore residents with technical training and computer skills so that they can earn a living in the current market through the East Baltimore Technology Resource Center, a new community entity [3].

This action by Johns Hopkins University was the realization that:

"Even though the 'best care in the world' may literally be next door, poor urban residents experience some of the worst health conditions, live in some of the least healthy environments, and have some of the worst health indices of any population group in the nation–in some instances comparable to those found in developing nations" [3].

Nearly all AMCs make some effort to provide healthcare access to the medically needy. Can all AMCs do what Johns Hopkins University did? Maybe not, but, nonetheless, every AMC should make an ongoing, informed, intentional effort to direct a certain amount of resources/effort toward improving healthcare access or correcting social determinants affecting a people's health or ability to access healthcare. If there are no resources available, then an effort should be made to acquire the necessary resources. Each AMC and their area partners can decide what their local priorities should be. Interventions may be broad or narrow, i.e., if a health system cannot provide an entire primary care program, they may wish to target less comprehensive projects such as providing vaccines, nutrition education, or access to tertiary services. A cooperative effort by AMCs and their partners can help identify targets and strategies within defined financial parameters, i.e., pick what you can do and how you can do it with the money you have available. AMCs have a moral obligation for such actions.

Action is needed to secure the health of the body and soul. Action is needed to secure the mutual well being of individuals and the preservation of a "normal" society where safety, education, economic opportunity, and access to healthcare would have at least some small chance to flourish. In short, AMCs must be the leaders in this effort. AMCs must use their power, through speech and action in the space of appearance, to secure the entrance of the powerless into the space of appearance so that healthcare access is available to all members of society.

## Conclusion

Once healthcare access is a right difficulties will invariably ensue. Do the elderly receive liver transplants? Can a morbidly obese person receive a reoperation for coronary artery bypass grafting? Can a diabetic receive a second kidney transplant? How much is enough healthcare access? Will healthcare delivery become a tiered process? Are we wise enough to make the necessary and correct decisions? Who will be society's proxy to make such decisions? There are no clear answers.

Some may think these decisions simply cannot be made. The argument presented here begs the opposite. Situations such as those found in East Baltimore, MD were amenable to interventions. The conditions regarding the ability of the medically needy to access healthcare can be resolved. As Hannah Arendt insightfully reminded us:

"The new always happens against the overwhelming odds of statistical laws and their probability, which for all practical, everyday purposes amounts to a certainty; the new therefore always appears in the guise of a miracle. The fact that man is capable of action means the unexpected can be expected from him, that he is able to perform what is infinitely improbable" [17].

## Competing interests

The author(s) declare that they have no competing interests.

## Authors' contributions

The author is responsible for the entire paper.
